# Metabolic dysfunction-associated steatotic liver disease and cardiovascular risk factors in rheumatoid arthritis

**DOI:** 10.1007/s10067-025-07364-5

**Published:** 2025-02-17

**Authors:** A. N. Saidi, W. B. Theel, B. Burggraaf, A. J. van der Lelij, D. E. Grobbee, J. D. van Zeben, E. van der Zwan-van Beek, S. P. Rauh, M. Castro Cabezas

**Affiliations:** 1 Department of Internal Medicine, Centre of Endocrinology, Diabetes and Vascular Medicine, Franciscus Gasthuis & Vlietland, Rotterdam, the Netherlands; 2 Department of Internal Medicine, Erasmus University Medical Center, Rotterdam, the Netherlands; 3 Obesity Center CGG, Franciscus Gasthuis & Vlietland, Rotterdam, the Netherlands; 4 Julius Center for Health Science and Primary Care, University Medical Center Utrecht, Utrecht, the Netherlands; 5 Julius Clinical, Zeist, the Netherlands; 6 Department of Rheumatology, Franciscus Gasthuis & Vlietland, Rotterdam, the Netherlands; 7 Department of Clinical Chemistry, Franciscus Gasthuis & Vlietland, Rotterdam, the Netherlands; 8 Department of Science, Franciscus Gasthuis & Vlietland, Rotterdam, the Netherlands

**Keywords:** Chylomicron remnants, FIB-4, Liver fibrosis, MASH, MASLD

## Abstract

**Objectives:**

Rheumatoid arthritis (RA) is a chronic autoimmune disease linked with metabolic dysfunction-associated steatotic liver disease (MASLD), which may increase cardiovascular (CV) risk. This study explores the association between liver fibrosis, assessed by the Fibrosis-4 (FIB-4) index, and CV risk factors in RA patients.

**Methods:**

Cross-sectional data from the Franciscus Rheumatoid Arthritis and Cardiovascular Intervention Study (FRANCIS), a randomized, cardiovascular single center, intervention study involving RA patients without cardiovascular disease (CVD) or type 2 diabetes (T2DM), were analyzed. Liver fibrosis was assessed using FIB-4, with a cut-off point of ≥ 1.3 to define high fibrosis risk, and its relationship with CV risk factors, medication use, and subclinical atherosclerosis, measured by carotid intima-media thickness (cIMT), was evaluated.

**Results:**

Among 326 patients (68.4% female, age 53 ± 11 years, BMI 26.5 ± 4.5 kg/m^2^), those with high FIB-4 (*n* = 49) had higher cIMT (*p* = 0.002), apolipoprotein B48 (*p* = 0.04), systolic blood pressure (*p* = 0.007), alkaline phosphatase (*p* = 0.002), and anti-CCP levels (*p* = 0.02). High FIB-4 was associated with lower leukocyte count and complement component 3. Statin use was linked to higher FIB-4 (OR = 4.49, *p* = 0.014), while hydroxychloroquine use was associated with lower FIB-4 (OR = 0.11, *p* = 0.004). Disease activity scores did not differ between low and high FIB-4 groups.

**Conclusions:**

Elevated FIB-4 in RA patients is associated with increased cIMT, higher blood pressure, and elevated atherogenic remnants. Incorporating FIB-4 measurements into routine clinical care for RA populations could effectively identify individuals at the highest CV risk, enabling the implementation of more intensive CV risk management strategies.

**Table:** 

**Key Points** *• RA patients with liver fibrosis have higher cIMT, indicating greater risk of atherosclerosis.* *• RA patients with liver fibrosis show accumulation of circulating atherogenic chylomicron remnants, contributing to atherogenesis.* *• HCQ may provide a protective effect against liver fibrosis in RA patients.*

**Supplementary Information:**

The online version contains supplementary material available at 10.1007/s10067-025-07364-5.

## Introduction

Rheumatoid arthritis (RA) is a chronic autoimmune and inflammatory disease which can lead to significant disability and reduced quality of life [1]. Beyond joint involvement, RA has notable extra-articular manifestations, impacting the lungs, skin, eyes, heart, kidneys, nervous system, and gastrointestinal tract [2, 3]. One potential clinical outcome of RA is liver involvement, which can manifest as autoimmune biliary disease, abnormal liver test results, or metabolic dysfunction-associated steatotic liver disease (MASLD) [4–6]. MASLD is characterized by excess fat accumulation in the liver along with at least one cardiometabolic risk factor, in the absence of secondary causes of hepatic steatosis or excessive alcohol consumption [7]. Approximately one-third of RA patients are affected by MASLD [8, 9], which can range from simple steatosis to more severe forms like metabolic dysfunction-associated steatohepatitis (MASH), potentially advancing to cirrhosis and hepatocellular carcinoma (HCC) [10]. Furthermore, MASLD is associated with an increased risk for CVD [11].

Treatment with disease-modifying antirheumatic drugs (DMARDs) may also contribute to the risk of MASLD in RA. Methotrexate (MTX), the most frequently used DMARD for RA, has been linked to various hepatic side effects, including elevated liver enzymes and an increased risk of liver fibrosis, especially with long-term use or higher doses [12, 13]. Other RA medications, such as biologicals, have also been associated with hepatotoxicity, although the extent of their contribution to liver fibrosis remains unclear [14]. Given the widespread use of these medications in RA management, it is crucial to evaluate their potential impact on liver health, particularly on the progression of liver fibrosis.

RA is closely linked to cardiovascular disease (CVD), primarily due to the combined effects of systemic inflammation, potentially causing harm to the arterial wall, and the presence of traditional CV risk factors, which are often undertreated in this population [15]. Patients with RA have a 1.5-fold increased risk of CVD and experience higher rates of CVD-related morbidity and mortality compared to the general population [16, 17]. The increased risk of premature mortality in RA patients is attributed to both persistent inflammation and inadequate management of traditional CV risk factors [18–22].

In addition to traditional CV risk factors, the carotid intima-media thickness (cIMT), a surrogate marker for subclinical atherosclerosis, has also been shown to be increased in RA patients [23, 24]. Recent studies also suggested that the increased CVD risk in RA may be associated with accumulation of atherogenic intestinal remnants carrying apolipoprotein (apo) B48 [25, 26].

Although CVD and MASLD are closely interconnected and inflammation plays a central role in both, this has not been studied in detail in a typical systemic inflammatory condition like RA. We hypothesized that the presence of MASLD, particularly liver fibrosis, a key determinant of MASLD prognosis, has been associated with increased CV risk [25].

While liver biopsy is considered the gold standard for diagnosing liver fibrosis, it is an invasive and costly procedure which is not routinely performed unless there is a clear clinical indication [27]. Therefore, we used the Fibrosis-4 (FIB-4) index, a non-invasive blood test recommended by the European Association for the Study of the Liver (EASL) guidelines, for the assessment of liver fibrosis [7, 28]. The aims of this study were to investigate the association between liver fibrosis, as estimated by the FIB-4, and CV risk factors, including hypertension, lipid levels, and cIMT in patients with RA. Additionally, it aimed to investigate the association between liver fibrosis and medication use in this patient population.

## Materials and methods

### Study design and study population

This is a post-hoc analysis of the Franciscus Rheumatoid Arthritis and Cardiovascular Intervention Study (FRANCIS), an open-label randomized controlled trial that aimed to investigate the effectiveness of a strict treatment of CV risk factors in RA [26, 29, 30]. The study population (*n* = 326) consisted of patients attending the outpatient clinic of the Department of Rheumatology at the Franciscus Gasthuis Hospital in Rotterdam, the Netherlands. Participants were included if they were less than 70 years of age and if they had been diagnosed with rheumatoid arthritis, defined according to the criteria of the American College of Rheumatology [31]. Patients were excluded if they had type 1 diabetes mellitus (T1DM) or T2DM at baseline, defined as a fasting glucose of > 7.0 mmol or HbA1c > 48 mmol/mol, or being treated for these conditions or if they had a history of CVD. The latter of which was defined as a prior myocardial infarction, cerebrovascular event, intermittent claudication, amputation due to peripheral artery disease (PAD), coronary artery bypass graft (CABG), or percutaneous transluminal coronary angioplasty (PTCA). Additionally, patients were excluded if they had kidney disease, defined as an estimated glomerular filtration rate (eGFR) of < 40 ml/min/1.73m^2^. The study was approved by the medical ethical committee of the Maasstad hospital in Rotterdam, the Netherlands, and was conducted in accordance with the Declaration of Helsinki (The Dutch Trial register, NTR3873; ABR no. NL32669.101.10) [32].

### Data collection

Baseline data was collected during outpatient clinic visits between 2009 and 2012. Blood samples were obtained after an overnight fast. In addition, anthropomorphic measurements (e.g., weight, height, blood pressure) were recorded. Variables included for medication use were methotrexate (MTX), NSAIDs, biologicals, statins, antihypertensives, leflunomide, hydroxychloroquine (HCQ), prednisone, and anti-TNF therapy. Furthermore, RA disease activity was evaluated using the Disease Activity Score (DAS28) wherein 28 joints are evaluated [33].

### Fibrosis-4 index

The FIB-4 was calculated according to the following formula: age (years) × AST(U/L)/platelet (PLT) (10^9^/L) × √ALT(U/L). Low hepatic fibrosis risk was defined as a FIB-4 < 1.3 and high fibrosis risk as a FIB-4 ≥ 1.3. The FIB-4 has been extensively validated in a broad spectrum of MASLD subjects and in RA patients [7, 28].

### Carotid intima media thickness

cIMT was measured by trained and experienced sonographers using the ART-LAB (Esaote, Italy) as described earlier [29]. Using B-mode ultrasounds which produced two echogenic lines, ultrasound images were made of the far wall of each common carotid artery (CCA). These lines represented the overall thickness of the media and intima layers of the arterial wall. Atherosclerotic plaque was defined as a lesion with a focal cIMT ≥ 1.0 mm.

### Laboratory measurements

In each patient, a standard set of laboratory measurements was performed at the Department of Clinical Chemistry of the Franciscus Gasthuis hospital. Blood samples were drawn following the standard procedures. Liver enzymes and renal tests as well as total cholesterol, HDL-C, glucose, C-reactive protein (CRP), and triglycerides (TGs) were measured using DxC analyzers (Beckman Coulter, United States) or Synchron LX20 (Beckman Coulter, United States) [34, 35]. LDL-C was measured using the Friedewald formula if TGs were below 4.00 mmol/L. Complement C3 levels were measured using the Atellica® Solution (Siemens Healthineers, Germany). Furthermore, using an ELISA, apoB48 serum levels were quantified as previously described [36].

### Statistical analysis

Data are presented as mean ± standard deviation (SD), unless otherwise specified. Normally distributed variables were analyzed using the independent *T*-test, while non-normally distributed parameters were assessed with the Mann–Whitney *U* test or Kruskal–Wallis test. Correlation analyses were conducted using Pearson’s or Spearman’s correlation coefficients, where appropriate. Multivariate binary logistic regression was performed with FIB-4 categories (low vs. high) as the dependent variable and medication use as the independent variables. Chi-square tests were also conducted. A multiple regression analysis was performed to investigate the association between FIB-4 (low vs. high) and cIMT adjusting for hypertension and lipid levels. All statistical analyses were performed using IBM SPSS Statistics version 28.0.0.0 (IBM SPSS Statistics, New York, United States), with statistical significance set at a *p*-value < 0.05.

## Results

**Fig. 1:**
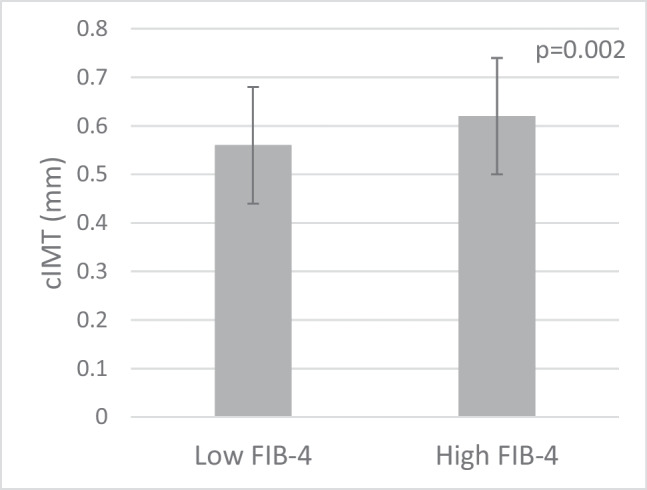
Mean carotid intima media thickness (cIMT) in the low FIB-4 versus high FIB-4 group; 0.57 mm ± 0.12 in the low FIB-4 group versus 0.62 mm ± 0.12 in the high FIB-4 group (*p* = 0.002)

The study population included 223 women and 103 men, with an average age of 53 years (± 11) and an average BMI of 26.5 kg/m^2^ (± 4.5). The majority of the cohort had a low FIB-4 score (*n* = 277, 85.0%). Patients with an elevated FIB-4 score were significantly older and had higher systolic blood pressure, higher apoB48 concentrations, higher cIMT values, higher alkaline phosphatase, and higher anti-cyclic citrullinated peptide (anti-CCP) concentrations (Table 1; Fig. 1). In addition, they had statistically significant lower BMI, as well as lower levels of thrombocytes and a lower leukocyte count, particularly neutrophils and lymphocytes, and lower levels of complement C3. There was no difference in disease activity score between groups (Table 1).

**Table 1:** Total group (*n* = 326). Baseline characteristics in the low FIB-4 versus high FIB-4 groups. *p*-value is for comparison between FIB-4 groups. Data are expressed as mean ± standard deviation, median (IQR), or *n* (%), as appropriate

	Low FIB-4 *N* = 277	High FIB-4 *N* = 49	*p*-value
**Demographic characteristics**			
Age (years)	52 ± 11	62 ± 7.2	< 0.001
Sex (m/f)	83/194 (30/70)	20/29 (41/59)	0.132
Smoking (no/yes)	207/55 (79/21)	39/9 (81/19)	0.724
**Clinical characteristics**			
Body mass index (kg/m^2^)	26.7 ± 4.6	25.2 ± 3.6	0.031
Systolic blood pressure (mmHg)	131 ± 18	139 ± 22	0.007
Diastolic blood pressure (mmHg)	78.9 ± 10	81 ± 12	0.142
Total cholesterol (mmol/L)	5.4 ± 1.1	5.6 ± 1.1	0.224
HDL-C (mmol/L)	1.5 ± 0.41	1.6 ± 0.43	0.069
LDL-C (mmol/L)	3.4 ± 0.94	3.4 ± 0.90	0.537
Triglycerides (mmol/L)	1.04 (0.72–1.55)	1.05 (0.76–1.49)	0.757
ApoAI (g/L)	1.68 ± 0.37	1.73 ± 0.37	0.441
Apo B (g/L)	1.00 ± 0.26	1.00 ± 0.25	1.000
Apo B48 (mg/L)	8.2 (5.0–12.1)	9.6 (6.8–15.5)	0.036
Fasting glucose (mmol/L)	5.5 ± 0.54	5.5 ± 0.63	0.350
HbA1c (mmol/mol)	35.3 ± 4.5	35.1 ± 3.9	0.839
CRP (mg/L)	3.0 (1.0–6.0)	2.0 (1.0–5.0)	0.181
Hemoglobin (g/dL)	8.5 ± 0.8	8.7 ± 0.5	0.002
Thrombocytes (10^9^/L)	255 ± 63.2	172 ± 30.9	< 0.001
Leukocyte counts (10^9^/L)	6.7 ± 2.0	5.2 ± 1.5	< 0.001
Neutrophils (10^9^/L)	4.1 ± 1.7	3.1 ± 1.2	< 0.001
Lymphocytes (10^9^/L)	1.8 (1.4–2.28)	1.5 (1.1–1.75)	0.024
Monocytes (10^9^/L)	0.55 ± 0.19	0.49 ± 0.18	0.057
Eosinophils (10^9^/L)	0.16 ± 0.11	0.16 ± 0.16	0.979
Basophils (10^9^/L)	0.03 ± 0.05	0.02 ± 0.04	0.128
Complement C3 (g/L)	1.19 ± 0.23	1.08 ± 0.18	< 0.001
Complement C4 (g/L)	0.21 ± 0.06	0.19 ± 0.05	0.053
Alkaline phosphatase (U/L)	70.5 ± 23.1	83.1 ± 38.2	0.002
Gamma-GT (U/L)	21 (16–29)	21 (16–32)	0.399
Creatinine (µmol/L)	69.5 ± 14.7	73.3 ± 13.9	0.092
Microalbumin (mg/L)	7.0 (5.0–14)	7.0 (5.0–15)	0.249
Uric acid (mmol/L)	0.29 (0.25–0.34)	0.32 (0.26–0.36)	0.878
TSH (mU/L)	1.9 (1.3–2.7)	2.0 (1.3–3.8)	0.221
Anti-CCP (U/mL)	0.64 ± 0.48	0.80 ± 0.41	0.027
DAS28CRP	2.5 ± 1.1	2.2 ± 0.93	0.171
DAS28BSE	2.6 ± 1.3	2.4 ± 0.98	0.287
ALAT (U/L)	16 (13—20)	23 (19.5—27)	< 0.001
ASAT (U/L)	25 (19–32)	27 (20–35)	0.086
FIB-4	0.73 ± 0.28	1.75 ± 0.45	< 0.001
**Medication use**			
Antihypertensives	43 (15.6)	10 (20.4)	0.405
Statins	9 (3.3)	6 (12.2)	0.015
Leflunomide	3 (1.1)	2 (4.1)	0.166
Hydroxychloroquine	71 (25.8)	2 (4.1)	< 0.001
Anti-TNF	97 (35.3)	19 (38.8)	0.747
Prednisone	34 (12.4)	5 (10.2)	0.814
Methotrexate	206 (74.9)	38 (77.6)	0.589
NSAID	120 (43.6)	20 (40.8)	0.755

*HDL-C* high-density lipoprotein cholesterol, *LDL-C* low-density lipoprotein cholesterol, *remnant-C* remnant cholesterol, *apoAI* apolipoprotein AI, *apoB* apolipoprotein B, *apoB48 apolipoprotein B48*, *HbA1c* hemoglobin A1C, *CRP* C-reactive protein, *Gamma-GT* gamma-glutamyl transferase, *TSH* thyroid-stimulating hormone, *anti-CCP* anti-cyclic citrullinated peptide, *DAS28CRP* Disease Activity Score 28-joint count with C-Reactive Protein, *DAS28BSE* Disease Activity Score 28-joint count with Erythrocyte Sedimentation Rate, *ALT* alanine aminotransferase, *AST* aspartate aminotransferase, *FIB-4* Fibrosis-4 index

Correlation analyses showed significant positive associations between FIB-4 and systolic blood pressure (rho = 0.301, *p* < 0.001), cIMT (rho = 0.409, *p* < 0.001), total cholesterol (rho = 0.212, *p* < 0.001), LDL-C (rho = 0.190, *p* < 0.001), and apoB48 (rho = 0.141,* p* = 0.011). In contrast, negative correlations were found between FIB-4 and complement C3 (rho =  − 0.161; *p* = 0.004) and leukocyte count (rho =  − 0.399, *p* < 0.001). There was no statistically significant correlation between FIB-4 and triglycerides (rho = 0.060, *p* = 0.278).

Logistic regression analysis showed that statin use was associated with high FIB-4, while HCQ use was associated with lower FIB-4 (Table 2). To further explore these associations, we conducted univariate logistic regression analyses for each medication separately (Supplementary Table S1). These analyses confirmed that statin use was significantly associated with high FIB-4, while HCQ use was significantly associated with lower FIB-4. No significant associations were observed for antihypertensives, leflunomide, anti-TNF agents, prednisone, MTX, NSAIDs, or other biologics (all *p* > 0.05). In terms of medication use, 12.2% of the patients in the high FIB-4 group used statins, compared to only 3.3% in the low FIB-4 group (Table 1). Similarly, HCQ use showed a statistically significant difference between the two groups, with 4.1% of the patients in the high FIB-4 group using HCQ, compared to 25.8% in the low FIB-4 group. These associations were found independently of the use of other medications included in the analysis. No significant differences were found for other medications, including antihypertensives, leflunomide, anti-TNF agents, prednisone, MTX, NSAIDs, and biologicals (all *p* > 0.05) (Table 1).

**Table 2:** Logistic regression analysis of medication effects on FIB-4 (low versus high)

Variable	B	S.E	Wald	df	Sig	Exp(B)	95% CI for Exp(B)
Constant	− 1.826	0.461	15.686	1	< 0.001	0.161	
Antihypertensives	0.257	0.423	0.370	1	0.543	1.293	0.565–2.962
Statins	1.501	0.612	6.019	1	0.014	4.488	1.352–14.890
Hydroxychloroquine	− 2.176	0.749	8.436	1	0.004	0.113	0.026–0.493
Anti-TNF	− 0.070	0.343	0.041	1	0.839	0.933	0.476–1.828
Prednisone	− 0.265	0.536	0.244	1	0.621	0.767	0.269–2.193
Methotrexate	0.403	0.420	0.921	1	0.337	1.497	0.657–3.411
NSAID	− 0.096	0.335	0.082	1	0.774	0.909	0.472–1.750

Higher FIB-4 scores are associated with increased cIMT. This relationship persists even when adjusting for potential confounders, including systolic blood pressure and LDL-C (Table 3). While adjustments were also made for HDL-C and diastolic blood pressure, the linearity with these variables was weaker than that of those included in the final model.

**Table 3:** Multiple regression analysis of cIMT and FIB-4, adjusted for confounding variables

Variable	*B*	95% CI	*p*-value
Model 1	0.055	0.021–0.089	0.002
Model 2	0.037	0.005–0.068	0.024

Model 1, crude model; model 2, adjusted for systolic blood pressure and LDL-C

## Discussion

The results from the current study have extended the findings from our previous observations reporting on the association between RA and CV risk factors, by also showing an association with liver fibrosis as estimated by FIB-4 [25, 26, 37]. The findings of the current study showed that RA patients with a high FIB-4 have higher cIMT values, even after adjusting for potential confounders, reinforcing the notion that liver fibrosis may independently contribute to CV risk in RA patients. This underscores FIB-4’s potential as a marker for both liver health and CV risk stratification in RA. Additionally, high systolic blood pressure was found to be significantly associated with increased cIMT, regardless of FIB-4 levels. This highlights the importance of managing blood pressure in RA patients, particularly in those with liver fibrosis, to reduce CV risk. There was no difference in disease activity score between those with low and high FIB-4, suggesting that liver fibrosis, as measured by FIB-4, may be independent of overall RA disease activity, which highlights the need to consider additional factors when assessing liver health in RA patients.

Previously, we reported elevated apoB48 concentrations in RA patients compared to controls [25, 26]. The present study expands that observation by also demonstrating an association with liver fibrosis. It is well known that chylomicrons and their remnants can directly infiltrate intact endothelium, become trapped, and induce an inflammatory response along with the formation of foam cells. This process plays a crucial role in the initiation and progression of atherosclerosis [37, 38]. This mechanism may explain the increased cIMT observed in RA patients with liver fibrosis. While the relationship between cIMT and liver fibrosis is evident, the pathophysiology of the accumulation of chylomicron remnants remains unclear. A key factor in the clearance of chylomicron remnants is a receptor-mediated pathway in the liver. Liver fibrosis could disrupt this process by reducing the number or function of these receptors. One could speculate that this impairment may lead to higher levels of circulating chylomicron remnants due to reduced hepatic clearance. Another important consideration is that RA patients with high FIB-4 levels also were more frequently on statins. Normally, statin use increases LDL-receptor expression, thereby enhancing the clearance of lipoproteins. This could either mean that these subjects had already been identified as having a higher CV risk and were therefore on appropriate treatment or that liver fibrosis might reduce statin effectiveness by impairing LDL-receptor activity, thus hindering the clearance of atherogenic lipoproteins despite statin treatment. At this stage, there is no clinical or experimental evidence supporting the latter hypothesis.

Another factor that may contribute to liver complications in RA is the presence of anti-CCP antibodies. While these antibodies are well-known for targeting various cell types in RA, their impact on liver cells is less understood. Recent laboratory studies have revealed that anti-CCP antibodies can influence hepatocytes by triggering the release of inflammatory proteins, impairing fat and glucose metabolism, and ultimately causing liver damage and apoptosis [39]. This mechanism could potentially explain the increased anti-CCP levels observed in patients with high FIB-4 scores.

Our study demonstrated a significant association between HCQ use and lower FIB-4 scores, a finding consistent with recent research suggesting that HCQ may have a protective effect against the development of MASLD [40]. HCQ is known to influence adiponectin levels, which play a key role in regulating metabolic processes. Higher adiponectin levels, linked to HCQ use, are associated with reduced systemic inflammation and improved insulin sensitivity, both of which can help mitigate liver damage [41]. This may explain the lower FIB-4 scores observed in patients using HCQ. However, it is important to consider that this association may be confounded by indication The protective effect of HCQ against liver fibrosis observed in this study may, in part, be attributed to its use in patients with lower RA disease activity. As low disease activity is inherently associated with reduced systemic inflammation, it is possible that the observed benefit reflects better-controlled RA rather than a direct pharmacological effect of HCQ. Given these considerations, the findings need to be interpreted with caution, and further studies are warranted to explore these potential mechanisms and establish the independent role of HCQ in liver fibrosis prevention.

Moreover, in our study, we did not find any significant associations between MTX use and liver fibrosis. Similarly, a recent multicenter study involving around 1000 patients with RA or psoriasis also found no association between the cumulative dose or duration of MTX exposure and liver fibrosis [42]. Although MTX has been previously linked to hepatotoxicity and an increased risk of liver fibrosis [13, 43], these newer findings suggest that the actual risk may be lower than previously believed. This discrepancy underscores the need for further research to clarify MTX’s impact on liver health and to better understand the factors that may influence these outcomes.

The lower inflammatory markers observed in patients with high FIB-4 levels could be attributed to the effects of advanced liver fibrosis. Advanced liver fibrosis often leads to portal hypertension and resultant hypersplenism, causing the sequestration of leukocytes and thrombocytes in the spleen, thereby reducing their levels in the bloodstream. Alternatively, the inverse relationship between leukocyte count and FIB-4 may also be attributed to MTX use, which can cause myelosuppression and reduce leukocyte counts, often leading to neutropenia, anemia, and thrombocytopenia [43, 44].

Additionally, in contrast with previous studies, our study did not find significant associations between glucocorticoid use and liver fibrosis [40, 45]. This may be due to differences in glucocorticoid dosage, treatment duration, or comorbidities within our cohort. Further research with larger samples and more detailed data on glucocorticoid use is needed to clarify its potential impact on liver fibrosis in patients with RA.

This study had some limitations. Firstly, the cross-sectional design limits our ability to determine causality and observe changes over time. Longitudinal studies are needed to explore these aspects further. Secondly, while FIB-4 provides valuable insights, it is not the gold standard for liver fibrosis assessment; liver biopsy remains the preferred method. Additionally, only a small portion of the study population had a high FIB-4. Another limitation is the lack of data on cumulative steroid use. As this information was not collected prospectively, it was not feasible to include it in our analysis. However, given the primary focus of this study on the associations between liver fibrosis and CVD risk factors, as well as medication use in patients with RA, we believe the absence of this data is unlikely to have impacted the validity of our key findings though it is an important consideration for future research. Moreover, some patients in this study were prescribed medications such as HCQ, prednisolone, and NSAIDs, which are not currently recommended as first-line treatments for rheumatoid arthritis. These prescriptions likely reflect individual clinical decisions, including intolerance or contraindications to recommended therapies like methotrexate, or historical prescribing practices. Such variations highlight the complexity of managing rheumatoid arthritis in real-world settings and should be considered when interpreting the study’s findings.

In conclusion, RA patients with a high FIB-4 showed increased cIMT, indicating higher CV risk. This relationship appears to be influenced by hypertension and the accumulation of intestinal atherogenic chylomicron remnants. The observed inverse relationship between FIB-4 with systemic inflammatory markers suggests a complex interaction that warrants further research. Hydroxychloroquine may have the potential to offer some protective effect against liver fibrosis in RA patients, though further research is needed to confirm this finding.

## Supplementary Information

Below is the link to the electronic supplementary material.

Supplementary file1 (DOCX 18 KB)

## Data Availability

The datasets generated during and/or analyzed during the current study are available from the corresponding author on reasonable request.
