# Supplementary material for: Metabolic dysfunction-associated steatotic liver disease and cardiovascular risk factors in rheumatoid arthritis
Source: Clin Rheumatol. 2025 Feb 17;44(4):1485–92. doi: 10.1007/s10067-025-07364-5 (PMC11993437; doi:10.1007/s10067-025-07364-5)
Supplement: Supplementary file 1 — Supplementary file1 (DOCX 18 KB) [file 10067_2025_7364_MOESM1_ESM.docx]

**Table S1.** Univariate logistic regression analysis of medication effects on FIB-4 (low versus high)

| Variable | B | S.E. | Wald | df | Sig. | Exp(B) |
| --- | --- | --- | --- | --- | --- | --- |
| Antihypertensives | 0.325 | 0.391 | 0.688 | 1 | 0.407 | 1.383 |
| Statins | 1.417 | 0.552 | 6.586 | 1 | 0.010 | 4.124 |
| Hydroxychloroquine | -2.102 | 0.735 | 8.175 | 1 | 0.004 | 0.122 |
| Anti-TNF | 0.145 | 0.319 | 0.205 | 1 | 0.650 | 1.156 |
| Prednisone | -0.216 | 0.506 | 0.183 | 1 | 0.669 | 0.805 |
| Methotrexate | 0.241 | 0.382 | 0.400 | 1 | 0.527 | 1.273 |
| NSAID | -0.129 | 0.315 | 0.167 | 1 | 0.683 | 0.879 |
